# Adipocytokine Profiles in Type 2 Diabetes Mellitus and Autoimmune Thyroid Disease: Associations With Metabolic and Hormonal Parameters

**DOI:** 10.33549/physiolres.935695

**Published:** 2026-06-01

**Authors:** Ivana JOCHMANOVÁ, Zbyněk SCHRÖNER, Štefan SOTAK, Marek FELŠÖCI, Darina PETRÁŠOVÁ, Miriam MITNÍKOVÁ, Izabela BERTKOVÁ, Hedviga WAGNEROVÁ, Ivica LAZÚROVÁ

**Affiliations:** 1 First Department of Internal Medicine, Louis Pasteur University Hospital and Pavol Jozef Šafárik University in Košice, Faculty of Medicine, Košice, Slovak Republic; 2 Department of Nuclear Medicine of Pavol Jozef Šafárik University in Košice, and Institute of Nuclear and Molecular Medicine, Košice, Slovak Republic; 3 Department of Geriatrics and Nursing, St. Luke Highly Specialized Institute of Geriatrics, Košice, Slovak Republic; 4 Laboratory of Research Bio-Models, Pavol Jozef Šafárik University in Košice, Faculty of Medicine, Košice, Slovak Republic; 5 Department of Laboratory Medicine, Louis Pasteur University Hospital, Košice, Slovak Republic; 6 Center of Clinical and Preclinical Research MEDIPARK, Pavol Jozef Šafárik University in Košice, Faculty of Medicine, Košice, Slovak Republic

**Keywords:** Type 2 diabetes mellitus, Autoimmune thyroid disease, Adipocytokines, Metabolic dysfunction

## Abstract

Type 2 diabetes mellitus (T2DM) and autoimmune thyroid disease (AITD) are complex disorders involving metabolic, immune, and hormonal dysregulation. Adipocytokines from adipose tissue influence metabolism, inflammation, and immune regulation. We investigated adiponectin, resistin, and visfatin in relation to thyroid, glucose, and metabolic parameters in adults with T2DM, AITD, both conditions, and healthy controls. A total of 385 adults were recruited. Clinical, anthropometric, and laboratory parameters were assessed, including glycemia, glycated hemoglobin (HbA1c), free thyroxine (fT4), thyroid-stimulating hormone (TSH), thyroid antibodies, and serum levels of adiponectin, resistin, and visfatin. Correlation and regression analyses were performed, including models adjusted for age, sex, and BMI. Adiponectin was higher in AITD than T2DM (p=0.012), resistin was higher in T2DM&AITD than controls (p=0.004), and visfatin was lower in T2DM than other groups (p<0.001). In T2DM&AITD, adiponectin correlated positively with age and fT4 and negatively with BMI (p=0.028, p=0.034). Resistin correlated inversely with TSH in AITD (p=0.033). Visfatin correlated inversely with thyroid volume in T2DM (p=0.003) and with waist circumference in AITD and T2DM&AITD (p=0.007, p=0.020). After adjustment, resistin remained higher in T2DM&AITD vs. controls (p=0.013) and visfatin and adiponectin remained lower in T2DM vs. controls (p=0.049 and p=0.045, respectively). This study highlights distinct adipocytokine profiles in individuals with metabolic and autoimmune thyroid conditions. Adiponectin, resistin, and visfatin may be potential biomarkers for disease severity and metabolic dysfunction. Further research is needed to elucidate the mechanistic pathways linking adipocytokines to the pathophysiology of T2DM and AITD.

## Introduction

Type 2 diabetes mellitus (T2DM) is a metabolic disease characterized by chronic hyperglycemia resulting from defects in insulin secretion and insulin resistance. Autoimmune thyroid disease (AITD), such as Hashimoto’s or Graves’ disease, is characterized by autoimmune reactions against thyroid antigens, enhanced lymphocytic infiltration, and the development of specific autoantibodies. It is well-established that insulin resistance and other metabolic abnormalities are associated with AITD in patients with type 2 diabetes mellitus (T2DM). Activation of the immune response may impair insulin sensitivity. The etiology and pathophysiology of AITD and T2DM remain unknown, but environmental, genetic, and hormonal factors contribute to their development [1]. However, obesity is a well-established risk factor associated with type 2 diabetes mellitus (T2DM). Thyroid hormone (TH) disturbances are also associated with changes in body weight as well as fat volume and distribution. THs play a key role in regulating body metabolism, including setting the resting metabolic rate, reducing energy expenditure, regulating thermogenesis in adipose tissue, and responding to catecholamines. Thyroid-stimulating hormone (TSH) receptors are present in adipose tissue, indicating their role in regulating adipocytokines [2].

Adipose tissue, once considered a passive storage depot for excess energy, is now recognized as an endocrine organ that secretes a diverse array of bioactive molecules [3], including adipocytokines such as adiponectin, resistin, and visfatin. These proteins have emerged as crucial in various physiological processes, including energy homeostasis, metabolism, inflammation, and immune response [4]. Abnormal levels of adipocytokines and other pro-inflammatory cytokines (tumor necrosis factor [TNF]-α, interleukins [IL]-1, -6, -10, monocyte chemoattractant protein, angiotensinogen, etc.) may activate monocytes, lead to local and systemic chronic low-grade inflammation, and enhance insulin resistance, further increasing the risk of T2DM. Ongoing research suggests that targeting inflammation may have therapeutic potential in managing metabolic disorders and their complications (reviewed in [5]).

The complex interplay between adipocytokines and metabolic disorders, such as T2DM and autoimmune conditions, such as AITD, underscores the importance of understanding their roles in disease pathogenesis. Adipocytokines can be practical markers of T2DM and metabolic syndrome severity or cancer. However, the role of adipocytokines in the etiology of AITD remains unclear [6,7]. Data on changes in adipocytokine levels during the development process of T2DM and AITD are inconsistent. Thus, in the present work, we aim to assess the association of three adipocytokines – adiponectin, resistin, and visfatin – with thyroid, glucose, and metabolic status in patients with T2DM, AITD, a combination of both diseases, and in control subjects without these diseases.

Adiponectin has been extensively studied in relation to obesity, insulin resistance, and cancer [8–13]. It is well-established for its association with obesity and thyroid diseases, making it a focal point in current research [12]. On the other hand, resistin has garnered attention due to its involvement in insulin resistance and diabetes mellitus [14]. Studies have shown that resistin levels are elevated in individuals with T2DM compared to controls [15]. It has also been associated with AITD, potentially through its role in modulating immune responses [16]. Visfatin, another adipocytokine secreted by visceral fat, mimics insulin effects and possesses proinflammatory properties [17,18].

## Methods

Adult patients with T2DM, AITD, or a combination of both disorders, and healthy control subjects were recruited at the 1^st^ Department of Internal Medicine of Louis Pasteur University Hospital and Pavol Jozef Šafárik University, Faculty of Medicine, in Košice, and its outpatient offices for endocrinology and diabetology between January 2013 and May 2023. The study was approved by the Ethics Committee of Louis Pasteur University Hospital in Košice, Slovakia, and is in accordance with the 1964 Helsinki Declaration. Written informed consent was obtained from all study participants.

For the T2DM group, the inclusion criterion was T2DM without a history of or current thyroid disease or other endocrine conditions. Patients with AITD without prior or current prediabetes or diabetes mellitus (DM) were included in the second group. The third arm of the study consisted of subjects with established diagnoses of both T2DM and AITD. In the control group (CG) subjects, exclusion criteria included DM, thyroid disease or any other endocrine disease, chronic pancreatitis, positivity for anti-GAD antibodies, cancer, and glucocorticoid treatment. In the AITD and control groups, current T2DM was excluded based on medical history and available laboratory data (fasting plasma glucose and HbA1c); an oral glucose tolerance test was not routinely performed.

### Subject evaluation and biological samples procurement

All subjects included in the study underwent clinical and laboratory evaluation. Basic anthropometric, thyroid, and metabolic parameters, as well as thyroid gland morphology, were assessed. Anthropometric data included age, gender, height, weight, body mass index (BMI), waistline, and thyroid gland volume. BMI was calculated as weight (kg) divided by the square of height (m^2^). Volume, morphology, and pathomorphology of the thyroid gland were assessed using Esaote Technos MPx ultrasound, performed by the same ultrasonographer and calculated by the formula (aL × bL × cL × π/6) + (aR × bR × cR × π/6), where aL – the height of left thyroid lobe in mm, bL – width of left thyroid lobe in mm, cL – length of left thyroid lobe in mm, aR – the height of right thyroid lobe in mm, bR – width of right thyroid lobe in mm, cR – length of right thyroid lobe in mm.

Fasting whole blood samples were obtained from each patient, and levels of the following parameters were evaluated: glycemia, glycated hemoglobin (HbA1c), adipocytokines adiponectin, resistin, and visfatin, free thyroxine (fT4), serum TSH, serum thyroglobulin (aTg) and thyroid peroxidase (aTPO) antibodies. Glycemia was determined using photometric colorimetry, and HbA1c, fT4, TSH, aTg, and aTPO levels were measured by electrochemiluminescent immunochemistry. The ELISA method was used to quantify adipocytokine concentrations.

HbA1c was assessed using Diabetes Control and Complications Trial (DCCT)-aligned standardization; for diabetes diagnosis, the ADA threshold is HbA1c ≥6.5 % (ADA, 2010), noting that diagnostic cut-offs may differ across professional societies. Diagnostic criteria for primary hypothyroidism included serum TSH concentrations >4.2 mIU/l and low or normal levels of fT4. Diagnostic criteria for AITD included positive ultrasonography (USG) image (diffuse homogeneous or blotchy hypoechogenicity of thyroid tissue) and the presence of aTg and/or aTPO antibodies.

### Statistical analysis

Statistical analyses were conducted using JASP version 0.19.3.0 (JASP Team, Amsterdam, The Netherlands). Continuous variables were tested for normality using the Shapiro-Wilk test. Data are presented as the mean ± standard deviation (SD) for normally distributed variables, and as the median with range (IQR) for non-normally distributed variables. Categorical variables are expressed as absolute frequencies and percentages. Continuous variables are presented as median and range or as mean and standard deviation (SD), and categorical variables are expressed as frequency counts and percentages. Comparisons of the central tendencies of the different groups were performed using an unpaired two-tailed Student *t*-test or a Mann-Whitney U test for non-normally distributed parameters. A simple linear regression analysis and a correlation analysis, using the determination of Pearson’s (r) or, for non-normally distributed parameters, Spearman’s (ρ) correlation coefficient, were employed as appropriate to assess the associations between the variables. The differences between continuous parameters divided into more than two subgroups were calculated by one-way ANOVA, with Tukey or Dunn’s *post hoc* tests used as appropriate. A p-value of <0.05 was considered statistically significant. All numerical values are rounded to two decimal places.

In addition, multivariable linear regression models were used to compare adipocytokine concentrations between groups while adjusting for age, sex, and BMI. Because adiponectin, resistin, and visfatin were right-skewed, adipocytokines were natural log-transformed prior to modeling; zero values (resistin and visfatin) were replaced by half of the minimum positive value to enable log transformation. Model results are presented as exponentiated coefficients (ratios) with 95 % confidence intervals.

### Ethics approval

Ethics Committee of Louis Pasteur University Hospital in Košice, Slovakia, and is in accordance with the 1964 Helsinki Declaration. Written informed consent was obtained from all study participants. No animals were used as part of this study.

## Results

### Patients

**Fig. 1:**
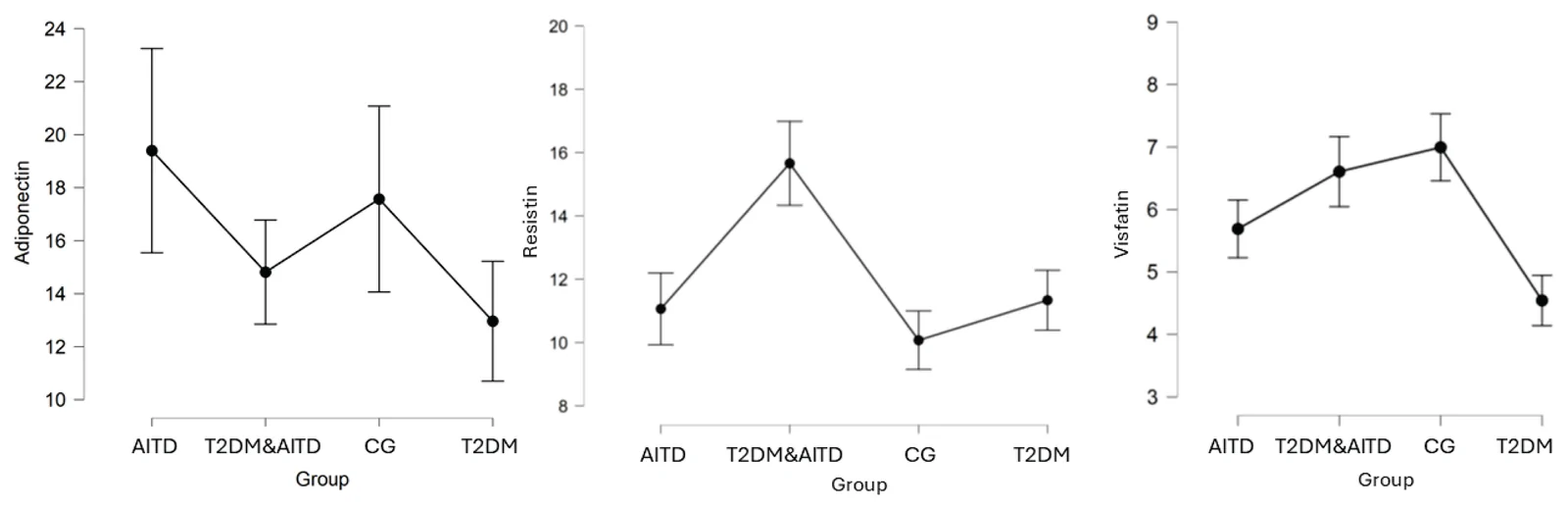
Serum levels of adipocytokines across study groups. Mean concentrations (± standard error of the mean) of adiponectin (left), resistin (middle), and visfatin (right) are shown for four groups: AITD (patients with autoimmune thyroid disease only), T2DM&AITD (patients with both autoimmune thyroid disease and type 2 diabetes mellitus), CG (healthy control subjects), and T2DM (patients with type 2 diabetes mellitus only). Data points represent group means connected by lines for visualization of trends. Error bars indicate standard errors. AITD – autoimmune thyroid disease, CG – control group, T2DM – type 2 diabetes mellitus, T2DM&AITD – type 2 diabetes mellitus and autoimmune thyroid disease.

Three-hundred eighty-five subjects were enrolled in the present study, 144 (37.40 %) males and 241 (62.60 %) females, who were divided into four groups: T2DM (98 [25.45 %] subjects), AITD (99 [25.71 %] subjects), T2DM&AITD (97 [25.20 %] subjects), and a CG (91 [23.64 %] subjects). The demographic parameters of the subjects in each group are shown in Table 1. There were significant differences between the groups in most measured parameters (Table 1). Levels of different adipocytokines in the studied groups are shown in Figure 1. *Post hoc* analysis revealed significantly higher adiponectin levels in AITD compared to T2DM subjects (p=0.012), and significantly higher resistin levels were found in T2DM&AITD subjects compared to the CG (p=0.004). Visfatin levels were significantly lower in the T2DM group compared to the AITD, T2DM&AITD, and CG groups (p=0.01, p=0.004, and p<0.001, respectively). Although all four studied groups were similar in size, there were significant differences between the number of males and females.

After adjustment for age, sex, and BMI in multivariable models, adiponectin was lower in the T2DM group vs. CG (adjusted ratio 0.80, 95 % CI 0.64–1.00; p=0.045), resistin remained higher in T2DM&AITD vs. CG (adjusted ratio 1.44, 95 % CI 1.08–1.93; p=0.013), and visfatin remained lower in T2DM vs. CG (adjusted ratio 0.63, 95 % CI 0.39–1.00; p=0.049; Table 3). Adjusted geometric means from these models are presented in Table 4.

Given the predominance of women and the median age in the sixth decade, menopausal status is a potential modifier of adipocytokine profiles; however, menopausal status was not systematically recorded and therefore could not be analyzed.

### Correlations of adipokines with age, anthropometric parameters, glucose, and thyroid status

We detected correlations between individual adipocytokines and certain evaluated parameters across the four studied groups (Table 2). In all groups, a positive correlation was observed between adiponectin levels and age. Resistin levels appear to increase with age in T2DM, AITD, and T2DM&AITD (p=0.003, <0.001, and 0.002, respectively; Table 2). A positive correlation of adiponectin with fT4 and a negative correlation with BMI were observed in the T2DM&AITD subjects p=0.028 and p=0.034, respectively; Table 2). A significant negative association between resistin and TSH levels was observed in the AITD group (p=0.033, Table 2). Surprisingly, we observed a weak negative correlation between visfatin and the volume of the thyroid gland in patients with T2DM without thyroid disease (p=0.003; Table 2). In T2DM group, positive correlation of visfatin with aTg has been observed (p=0.006; Table 2). We also detected a moderate negative correlation between visfatin and waist circumference in the AITD, T2DM, and T2DM&AITD groups (p=0.007, p=0.02, and p=0.02, respectively; Table 2) and a moderate negative correlation between visfatin and BMI (p=0.004) in the T2DM&AITD group. Adiponectin was weakly negatively correlated with HbA1c levels in T2DM group (p=0.036; Table 2). In CG we determined a moderate positive correlation between visfatin and TSH and adiponectin and TSH levels (p=0.010 and p=0.045, respectively; Table 2).

Several associations showed similar directions across subgroups but did not reach statistical significance within the single-disease groups, whereas they became significant in the T2DM&AITD group. This may reflect both increased statistical power within a more homogeneous phenotype of comorbidity and true biological differences in patients with coexisting metabolic and AITD; therefore, these findings should be interpreted cautiously until confirmed in adjusted multivariable analyses.

Comparison of the association between particular adipocytokines in the studied groups revealed a strong positive correlation between adiponectin and resistin in the AITD and T2DM&AITD groups (p<0.001, Table 2) and a weak correlation between the same adipocytokines (p=0.049, Table 2) in the T2DM group. Visfatin was weakly positively correlated with adiponectin in T2DM&AITD subjects (p=0.039, Table 2). A strong positive correlation was observed between visfatin and resistin in the T2DM, AITD, and CG groups (p<0.001, Table 2).

## Discussion

Several authors suggest that hypothyroidism may potentiate the production of adipocytokines. Our unadjusted group comparisons, shown in Table 1 and Figure 1, found gradually increasing mean adiponectin concentrations in subjects with T2DM vs. T2DM&AITD vs. CG and vs. AITD; however, significantly higher levels were found only in AITD subjects compared to T2DM. Resistin levels were slightly higher in AITD and T2DM groups than CG, but only in T2DM&AITD subjects were significantly higher than in those with T2DM and CG. In contrast, the average visfatin concentration was highest in CG subjects compared to the other three studied groups, with significantly lower levels found in T2DM subjects. In multivariable models adjusted for age, sex, and BMI (Table 3), adiponectin and visfatin remained lower in T2DM compared with CG (adjusted ratios 0.80 and 0.63; p=0.045 and p=0.049, respectively), while resistin remained higher in T2DM&AITD compared with CG (adjusted ratio 1.44; p=0.013).

### Adiponectin

Adiponectin is a protein that white adipose tissue (WAT) produces. It is a member of the soluble collagen superfamily with several isoforms. It is the most abundant adipokine in human blood. Adiponectin is involved in various biological functions, including regulation of insulin sensitivity, glucose uptake, lipid metabolism, and inflammation, and thus contributes to the pathogenesis of many diseases [19]. Adiponectin appears to have both pro-inflammatory and anti-inflammatory properties. Its low levels are associated with insulin resistance and decreased glucose tolerance, which can lead to liver injury. On the other hand, it has significant anti-inflammatory effects and participates in the oxidation of fatty acids in the liver and skeletal muscles, as well as in lipolysis and thermogenesis [20]. Plasma adiponectin levels are higher in females than in males, thus indicating a gender-dependent pattern [21]. In healthy individuals, adiponectin levels are negatively correlated with the amount of adipose tissue, particularly visceral fat [22]. In our study, we did not find any differences between adiponectin levels and waist circumference in any of the studied groups, but we observed a negative correlation between adiponectin concentration and BMI in T2DM&AITD subjects (Table 2). Low adiponectin levels were found in patients with T2DM [20,23–25]. A recent case-control study by Mir *et al*. [26] found significantly lower levels of serum adiponectin in T2DM patients compared to the control CG. In our cohort, we found significantly lower levels of adiponectin in T2DM subjects compared to those with AITD but not CG or T2DM&AITD subjects. In multivariable models adjusting for age, sex, and BMI, adiponectin was lower in T2DM compared with CG (p=0.045), whereas AITD did not differ from CG (p=0.794) (Table 3), suggesting that part of the unadjusted between-group contrast reflects differences in adiposity and demographic structure.

In the present cohort, adiponectin correlated positively with age across all groups (Table 2). This age-related rise has been reported in population studies and may reflect age-related changes in body composition (loss of skeletal muscle mass and relative fat redistribution), altered sex-steroid milieu (particularly after menopause), reduced clearance, or survivor bias. Given that age and menopausal status may confound adiponectin-metabolic associations, future analyses should assess whether the adiponectin-age relationship persists after adjustment for sex, BMI, and (where available) menopausal status or markers of sarcopenia.

Notably, mean adiponectin concentrations were highest in the AITD group (Table 1). Although adiponectin is generally considered protective in metabolic disease, very high levels have been linked to an ‘adiponectin paradox’ in some chronic conditions, where elevated adiponectin is associated with frailty, muscle wasting, and higher mortality risk [27,28]. In our study, this pattern could be influenced by older age, sex distribution, thyroid status, and unmeasured factors such as sarcopenia or menopause, and it warrants focused investigation in longitudinal and adequately adjusted analyses.

As mentioned above, THs are key players in regulating body metabolism. Moreover, they share some physiological actions with adiponectin, including reduction of adipose tissue *via* thermogenesis and increased lipid oxidation [29,30]. In animal experiments, it has been demonstrated that adiponectin gene expression is directly regulated by THs [31], and hyperthyroidism leads to an increase in adiponectin levels [32]. We did not find a positive or negative association of TSH or fT4 with adiponectin levels. Some works found a negative correlation between TSH and adiponectin in obese women [33], some, similarly to us, did not find any differences in adiponectin concentrations between hypo-, eu-, and hyperthyroid subjects [30,34–39]. Other authors observed that adiponectin levels correlate positively with fT4 and fT3 but not with TSH levels [40]. Yildiz *et al*. [41] showed that treatment with levothyroxine leads to an increase in adiponectin levels in patients with subclinical hypothyroidism. An increase in adiponectin concentrations was also reported after treatment and achieving the euthyroid status in hyperthyroid patients without brown adipose tissue (BAT), but not in those with BAT [42]. These discrepancies suggest that other mechanisms are also involved in regulating adiponectin and TH levels in humans.

### Resistin

Proinflammatory adipokine resistin (or adipose-tissue-specific secretory factor, ADSF) is a small cysteine-rich secretory protein produced in WAT with the aid of peripheral blood monocytes and other immune cells. Several studies in rodent models indicate that resistin is one of the key players in the development of metabolic syndrome, insulin resistance, inflammation, and immune regulation (reviewed in [43,44]). Resistin and leptin interact and regulate glucose metabolism and energy expenditure [44–46]. The highest levels of resistin were found in female gonadal tissue [44], thus, it is higher in females compared to males.

Besides T2DM and obesity, resistin is also linked to cardiovascular diseases, atherosclerosis, hypertension, malignancies, and arthritis [43]. A strong positive correlation between resistin levels and hypertension has been reported by Zhang *et al*. [47], and resistin is also associated with metabolic disease, increased coronary artery calcification [48], and aortic stiffness in patients with cardiovascular diseases [49]. It increases the levels of LDL-cholesterol and prevents its disintegration in the liver [50]. Moreover, resistin levels are inversely correlated with age, HDL-cholesterol, and physical activity and positively correlated with smoking and a sedentary lifestyle [51].

In terms of thyroid disease, a recent meta-analysis found significantly higher levels of resistin in patients with hyperthyroidism and subclinical hypothyroidism and a significant decrease of resistin after treatment of thyroid dysfunction. The association between resistin levels and hypothyroidism is not clear [44]. Yaturu *et al*. [40] observed significantly lower resistin levels in hypothyroid subjects compared to those with hyperthyroidism. In our study, resistin levels were slightly higher in AITD and T2DM groups than in CG, but only in T2DM&AITD subjects were significantly higher than in those with T2DM and CG (Table 1). Similar results were reported by Tabandeh *et al*. [52], who found that clinical thyroid dysfunction in T2DM subjects was associated with increased levels of resistin. A positive correlation between resistin and fT3 levels has been reported in the meta-analysis by Zhou *et al*. [44]. However, we did not observe such a correlation in any of our study subgroups. Notably, in the AITD subgroup, a negative correlation between resistin and TSH levels was observed (Table 2). In all but CG subjects, a positive correlation with adiponectin levels was present in our study (Table 2). Because we did not perform within-group multivariable analyses for these correlations, the resistin-TSH association should be interpreted cautiously as it may be confounded by age, sex, adiposity, and treatment-related factors.

### Visfatin

Visfatin possesses both cytokine-like extrinsic activity and an enzymatic intrinsic activity. Initially, it was discovered in the bone marrow and was named pre-B-cell colony-enhancing factor (PBEF). Later, it was found to have enzymatic properties of catalyzing the synthesis of nicotinamide mononucleotide (NMN), and it was renamed nicotinamide phosphoribosyl transferase (NAMPT). Visfatin/NAMPT exists in intra- and extracellular forms which exert various effects in cells. These include cell division/proliferation, differentiation, survival/apoptosis, life span, inflammation, cell metabolism and expenditure, responses of cells to environmental or oxidative stress, DNA integrity and repair, gene expression, mitochondrial health, and transcriptional and post-translational regulation. Moreover, extracellular visfatin plays a role in vascular remodeling and lipogenesis; it binds to insulin receptors, increases insulin sensitivity, lowers glucose levels, and enhances glucose uptake and transport [18]. Thus, visfatin is involved in NAD^+^-dependent signaling pathways, which are essential for various cellular biological functions and it is also involved in metabolic processes. Visfatin is expressed in adipose tissues, muscle, liver, immune cells, cardiomyocytes, and cardiac fibroblasts; it has been found in brain neuronal cells [18].

A possible association between visfatin and metabolic disorders such as obesity, diabetes, and insulin resistance has been suggested by many studies [53]. Increased serum visfatin levels have been found in various types of DM by some researchers [54–58], others did not find any association between DM and visfatin levels [59,60]; reviewed in [53]. In our study, lowest serum levels of visfatin were observed in T2DM subjects and were significantly lower than in other studied groups. Thus, our results suggest that rather lower than higher visfatin levels are associated with T2DM. This is in accordance with glucose-lowering effects of visfatin [61].

Our results showed visfatin levels negatively correlate with BMI in T2DM&AITD group and with waist circumference in AITD and T2DM&AITD groups. High levels of visfatin have been shown to be positively correlated with obesity and with the amount of visceral fat (reviewed in [20]).

These correlation findings are unadjusted and may be influenced by age, sex, adiposity, and treatment-related factors; therefore, they should be interpreted cautiously.

Visfatin plays a role in immune system regulation and inflammation, making it relevant to AITD. The relationship between visfatin and thyroid disorders has been examined in several studies and their results are conflicting. Higher levels of visfatin have been found in subjects with hypothyroidism (predominantly in Hashimoto’s thyroiditis) with a decrease in serum visfatin levels after adequate treatment of thyroid disease [62–65]. An inverse pattern between the alteration of serum visfatin and thyroid hormones was observed in the study of Tabandeh *et al*. [52]. A positive association between circulating visfatin with free thyroid hormones and aTPO in hypothyroidism has been reported by Sawicka-Gutaj *et al*. [66]. We did not observe such associations in our study, and there were no differences in visfatin levels between AITD, T2DM&AITD, and CG groups. However, like Ozkaya *et al*. [63], we observed a positive correlation between visfatin and TSH levels in CG.

### Clinical implications

From a clinical perspective, the observed adipocytokine patterns may help to refine phenotyping of patients with isolated metabolic disease, isolated AITD, and their coexistence. However, the effect sizes are modest and potentially confounded by age, sex, adiposity, menopausal status, smoking, and pharmacotherapy; therefore, adiponectin, resistin, and visfatin should currently be viewed as exploratory biomarkers rather than diagnostic tools. Future work should evaluate whether adipocytokines add incremental value beyond standard clinical variables (e.g., BMI, HbA1c, TSH/fT4, lipid profile) using multivariable models and discrimination/reclassification metrics.

## Limitations of the Study

While our study provides valuable insights into the relationship between adipocytokines, T2DM, and AITD, several limitations should be considered when interpreting the findings.

This study utilizes a cross-sectional design, which limits the ability to infer causal relationships between adipocytokine levels and the presence of T2DM and AITD. Longitudinal studies are needed to determine whether alterations in adipocytokine levels precede disease onset or result from metabolic and autoimmune dysregulation.

While we attempted to control for certain variables by excluding participants with cancer and those undergoing glucocorticoid treatment, other potential confounders, such as dietary habits, physical activity levels, smoking status, and medication use (e.g., metformin, levothyroxine), were not explicitly controlled for. These factors could influence adipocytokine levels and may have affected our results. In addition, menopausal status and sex-specific differences in adiposity distribution were not captured in sufficient detail to allow stratified or adjusted analyses.

The study population was predominantly female, particularly in the AITD groups, reflecting the known higher prevalence of AITD among women. However, this gender imbalance may have introduced bias, as sex hormones and metabolic differences between males and females influence adipocytokine levels. Future studies with a more balanced gender distribution are warranted.

The study did not stratify participants based on the severity or duration of T2DM or AITD, nor did it account for variations in glycemic control or thyroid hormone replacement therapy. These factors could significantly impact adipocytokine levels and should be considered in future research.

Although the overall cohort size was reasonable (N=385), dividing participants into four groups reduced the statistical power for subgroup analyses. Larger sample sizes in future studies may help improve the reliability of observed associations.

The study assessed adipocytokine levels at a single time point, preventing analysis of temporal changes in response to disease progression or treatment. Longitudinal studies would provide more comprehensive insights into how adipocytokine levels fluctuate over time and their role in disease pathophysiology.

Despite these limitations, our findings contribute to the growing body of knowledge regarding the interplay between adipose tissue-derived cytokines, metabolic dysregulation, and autoimmune disease. Further studies addressing these limitations will enhance our understanding of the role of adipocytokines in T2DM and AITD.

## Conclusions

This study provides novel insights into the associations between adipocytokines – adiponectin, resistin, and visfatin – and metabolic as well as thyroid-related parameters in individuals with T2DM, AITD, both conditions, and control subjects. Our findings suggest a potential role for adipocytokines as biomarkers for metabolic and autoimmune thyroid disorders. The observed associations between adipocytokines and metabolic parameters emphasize the need for further research on their mechanistic roles in disease pathogenesis. These findings warrant further exploration of adipocytokines as potential biomarkers and therapeutic targets in metabolic and autoimmune diseases.

## Figures and Tables

**Table 1:** Demographics, glucose and thyroid metabolism characteristics and adipokine values in studied groups and whole cohort.

	AITD	T2DM	T2DM&AITD	CG	p
*Males (N, %)*	16, 4.16	61, 15.84	19, 4.94	48, 12.47	<0.001
*Females (N, %)*	83, 21.56	37, 9.61	78, 20.26	43, 11.17	<0.001
*All (N, %)*	99, 25.71	98, 25.45	97, 25.20	91, 23.64	<0.001
*Age (years)* *	63 (19–65)	63 (41–65)	65 (27–65)	63 (20–65)	0.003
*BMI (kg/m* *^2^* *)* *	23.84 (19.1–46.9)	30.1, (18.3–57.78)	26.6 (20.7–49.7)	23.7 (12–40.15)	<0.001
*Height (cm)* *	168 (150–185)	171 (155–185)	166 (150–182)	171 (159–197)	<0.001
*Weight (kg)* *	68.0 (53.9–126.7)	85 (58–165.4)	71 (53.0–145.0)	71 (43.0–150.0)	<0.001
*Waist (cm)* *	76.0 (59.0–123.0)	96 (70–140)	82 (70.0–132.0)	82 (55–127)	<0.001
*Thyroid volume (ml)* *	9.78 (4.4–38.80)	13.15 (5.68–29.2)	9.74 (4.23–25.74)	11.76 (4.3–21.62)	<0.001
*TSH (mIU/l)* *	3.1 (0.047–129.70)	2.09 (0.02–14.03)	2.94 (0.18–111.62)	1.62 (0.25–6.16)	<0.001
*fT4 (pmol/l)* *	13.96 (3.20–27.78)	13.44 (7.69–34.86)	14.23 (3.2–22.07)	13.4 (5.6–23.99)	0.597
*aTg (kIU/l)* *	28.86 (5.0–4000.0)	12.32 (6.95–271.7)	24.2 (9.4–4000.0)	14.24 (0.9–78.58)	<0.001
*aTPO (kIU/l)* *	62.54 (5.0–2734.3)	10.92 (5.0–53.26)	23.45 (5.0–383.7)	10.61 (1.16–32.58)	<0.001
*Glycemia (mmol/l)* *	4.90 (3.4–6.9)	7.0 (3.8–26.2)	7.5 (3.9–26.1)	5.2 (2.8–7.1)	<0.001
*HbA1c (%)* *	5.3 (3.0–6.3)	6.9 (3.5–16.37)	6.7 (4.2–17.2)	5.2 (3.0–6.5)	<0.001
*Adiponectin (μg/ml)* †	20.97 (21.81)	13.55 (11.56)	14.81 (9.75)	17.60 (16.98)	0.012
*Resistin (ng/ml)* †	11.40 (11.52)	11.55 (9.68)	15.66 (13.06)	10.20 (8.84)	0.004
*Visfatin (ng/ml)* †	6.60 (5.06)	4.16 (3.56)	6.61 (5.53)	7.06 (5.15)	<0.001

* expressed as median (range),

† expressed as mean (SD).

AITD – autoimmune thyroid disease, aTg – anti-thyroglobulin antibodies, aTPO – anti-thyroid peroxidase antibodies, BMI – body mass index, CG – control group, fT4 – free thyroxine, HbA1c – glycated hemoglobin, T2DM – type 2 diabetes mellitus, T2DM&AITD – type 2 diabetes mellitus and autoimmune thyroid disease, TSH – thyroid stimulating hormone.

**Table 2:** Correlations between individual adipokines and age, parameters of glucose and thyroid metabolism in studied groups.

	Adiponectin	Resistin	Visfatin
*T2DM*			

*Age*	**ρ=0.395, p<0.001**	**ρ=0.299, p=0.003**	ρ =0.116, p=NS
*Thyroid volume*	ρ=−0.099, p=NS	ρ=−0.032, p=NS	**ρ=**−**0.298, p=0.003**
*fT* *_4_*	ρ=−0.028, p=NS	ρ=0.076, p=NS	ρ=0.178, p=NS
*TSH*	ρ=0.076, p=NS	ρ=−0.176, p=NS	ρ=−0.059, p=NS
*aTg*	ρ=−0.084, p=NS	ρ=0.181, p=NS	**ρ=0.276, p=0.006**
*aTPO*	ρ=0.009, p=NS	ρ=0.096, p=NS	ρ=−0.108, p=NS
*Gly*	ρ=−0.142, p=NS	ρ=−0.001, p=NS	ρ=−0.024, p=NS
*HbA1c*	**ρ=**−**0.212, p=0.036**	ρ =−0.165, p=NS	ρ=−0.104, p=NS
*BMI*	ρ=−0.070, p=NS	ρ=−0.080, p=NS	ρ=−0.134, p=NS
*Waist*	ρ=−0.014, p=NS	ρ=−0.072, p=NS	**ρ=**−**0.158, p=0.02**
*Adiponectin*		**ρ=0.200, p=0.049**	ρ=0.188, p=NS
*Resistin*			**ρ=0.344, p<0.001**

*AITD*			

*Age*	**ρ=0.495, p<0.001**	**ρ=0.421, p<0.001**	**ρ=0.297, p=0.003**
*Thyroid volume*	ρ=0.045, p=NS	ρ=−0.123, p=NS	ρ=−0.103, p=NS
*fT* *_4_*	ρ=0.021, p=NS	ρ=−0.097, p=NS	ρ=−0.076, p=NS
*TSH*	ρ=0.094, p=NS	**ρ=**−**0.215, p=0.033**	ρ=0.174, p=NS
*aTg*	ρ=0.168, p=NS	ρ=0.171, p=NS	ρ=0.107, p=NS
*aTPO*	ρ=0.066, p=NS	ρ=0.053, p=NS	ρ=0.098, p=NS
*Gly*	ρ=0.094, p=NS	ρ=0.078, p=NS	ρ**=**−0.065, p=NS
*HbA1c*	ρ=−0.015, p=NS	ρ=−0.056, p=NS	ρ =0.082, p=NS
*BMI*	ρ=−0.085, p=NS	ρ=−0.190, p=NS	ρ=−0.147, p=NS
*Waist*	ρ=−0.116, p=NS	ρ=−0.183, p=NS	**ρ=**−**0.270, p=0.007**
*Adiponectin*		**ρ=0.411, p<0.001**	ρ=0.118, p=NS
*Resistin*	**ρ=0.411, p<0.001**		**ρ=0.377, p<0.001**

*T2DM&AITD*			

*Age*	**ρ=0.530, p<0.001**	**ρ=0.312, p=0.002**	ρ=0.185, p=NS
*Thyroid volume*	ρ=0.144, p=NS	ρ=0.080, p=NS	ρ=0.171, p=NS
*fT* *_4_*	**ρ=0.224, p=0.028**	ρ=0.024, p=NS	ρ=0.026, p=NS
*TSH*	ρ=0.124, p=NS	ρ=−0.131, p=NS	ρ=0.009, p=NS
*aTg*	ρ=0.039, p=NS	ρ=0.071, p=NS	ρ=0.105, p=NS
*aTPO*	ρ=−0.039, p=NS	ρ=−0.010, p=NS	ρ=−0.048, p=NS
*Gly*	ρ=−0.092, p=NS	ρ=0.092, p=NS	ρ=0.043, p=NS
*HbA1c*	ρ=−0.165, p=NS	ρ=−0.071, p=NS	ρ=−0.034, p=NS
*BMI*	**ρ=**−**0.216, p=0.034**	ρ=−0.184, p=NS	**ρ=**−**0.291, p=0.004**
*Waist*	ρ=−0.188, p=NS	ρ=−0.158, p=NS	**ρ=**−**0.236, p=0.020**
*Adiponectin*		**ρ=0.424, p<0.001**	**ρ=0.210, p=0.039**
*Resistin*			ρ=0.185, p=NS

*CG*			

*Age*	**ρ=0.256, p=0.014**	ρ=0.009, p=NS	ρ=0.184, p=NS
*Thyroid volume*	ρ=−0.118, p=NS	ρ=0.063, p=NS	ρ=−0.118, p=NS
*fT* *_4_*	ρ=−0.014, p=NS	ρ=0.113, p=NS	ρ=0.178, p=NS
*TSH*	**ρ=0.210, p=0.045**	ρ=0.130, p=NS	**ρ=0.270, p=0.010**
*aTg*	ρ=0.119, p=NS	ρ=−0.064, p=NS	ρ=−0.054, p=NS
*aTPO*	ρ=0.188, p=NS	ρ=0.044, p=NS	ρ=0.104, p=NS
*Gly*	ρ=0.087, p=NS	ρ=−0.010, p=NS	ρ=0.029, p=NS
*HbA1c*	ρ=−0.004, p=NS	ρ=0.160, p=NS	ρ=0.165, p=NS
*BMI*	ρ=−0.146, p=NS	ρ=0.088, p=NS	ρ=0.161, p=NS
*Waist*	ρ=−0.086, p=NS	ρ=−0.021, p=NS	ρ=−0.049, p=NS
*Adiponectin*		ρ=0.095, p=NS	ρ=0.164, p=NS
*Resistin*			**ρ=0.485, p<0.001**

AITD – autoimmune thyroid disease, aTg – anti-thyroglobulin antibodies, aTPO – anti-thyroid peroxidase antibodies, BMI – body mass index, CG – control group, fT4 – free thyroxine, Gly – glycemia, HbA1c – glycated hemoglobin, T2DM – type 2 diabetes mellitus, T2DM&AITD – type 2 diabetes mellitus and autoimmune thyroid disease, TSH – thyroid stimulating hormone.

**Table 3:** Multivariable-adjusted differences in adipocytokine concentrations between clinical groups adjusted for age, sex, and BMI.

*Outcome*	Comparison (vs. CG)	N group	N CG	Adj. ratio	95 % CI	p
*Adiponectin*	AITD	99	91	1.03	0.83–1.27	0.794
*Adiponectin*	T2DM	98	91	0.8	0.64–1.00	**0.045**
*Adiponectin*	T2DM&AITD	97	91	0.89	0.72–1.11	0.308
*Resistin*	AITD	99	91	1.06	0.80–1.40	0.68
*Resistin*	T2DM	98	91	1.28	0.96–1.72	0.093
*Resistin*	T2DM&AITD	97	91	1.44	1.08–1.93	**0.013**
*Visfatin*	AITD	99	91	0.75	0.48–1.17	0.203
*Visfatin*	T2DM	98	91	0.63	0.39–1.00	**0.049**
*Visfatin*	T2DM&AITD	97	91	0.75	0.47–1.18	0.212

Multivariable linear regression models were fitted separately for adiponectin, resistin, and visfatin with Group (AITD, T2DM, T2DM&AITD) compared against CG (reference), adjusting for age (years), sex, and BMI (kg/m^2^). Outcomes were analyzed on the log scale to address right-skewness; results are presented as adjusted ratios (exponentiated regression coefficients) with 95 % CI and p-values. An adjusted ratio >1 indicates higher, and <1 lower, adipocytokine concentration relative to CG after covariate adjustment. Group sample sizes are reported. AITD – autoimmune thyroid disease, CG – control group, CI – confidence interval, T2DM – type 2 diabetes mellitus, T2DM&AITD – type 2 diabetes mellitus and autoimmune thyroid disease.

**Table 4:** Multivariable-adjusted adipocytokine levels by group (adjusted geometric means).

*Outcome*	Group	N	Adj. geom.	95 % CI
*Adiponectin*	CG	91	13.06	11.17–15.26
*Adiponectin*	AITD	99	13.44	11.57–15.61
*Adiponectin*	T2DM	98	10.37	8.84–12.14
*Adiponectin*	T2DM&AITD	97	11.63	10.01–13.50
*Resistin*	CG	91	6.95	5.64–8.56
*Resistin*	AITD	99	7.39	6.05–9.02
*Resistin*	T2DM	98	9	7.29–11.08
*Resistin*	T2DM&AITD	97	10.15	8.33–12.35
*Visfatin*	CG	91	4.28	3.10–5.90
*Visfatin*	AITD	99	3.2	2.35–4.37
*Visfatin*	T2DM	98	2.68	1.93–3.71
*Visfatin*	T2DM&AITD	97	3.19	2.35–4.35

Adjusted group estimates for adiponectin, resistin, and visfatin are presented as geometric means with 95 % CI, derived from the multivariable models adjusting for age, sex, and BMI. Estimates were calculated at the sample mean values of age and BMI and averaged across sexes according to the observed sex distribution. Values are back-transformed from the log scale to the original units. Group sample sizes are reported. AITD – autoimmune thyroid disease, CG – control group, T2DM – type 2 diabetes mellitus, T2DM&AITD – type 2 diabetes mellitus and autoimmune thyroid disease.
